# A Systematic Review of Ureteral Reimplantation Techniques in Endometriosis: Laparoscopic Versus Robotic-Assisted Approach

**DOI:** 10.3390/jcm13195677

**Published:** 2024-09-24

**Authors:** Stefano Di Michele, Silvia Bramante, Maurizio Rosati

**Affiliations:** 1 Division of Gynecology and Obstetrics, Department of Surgical Sciences, University of Cagliari, 09124 Cagliari, Italy; 2 Unit of Obstetrics and Gynecology, Santo Spirito Hospital, 65124 Pescara, Italy; silvia.bramante@asl.pe.it (S.B.); maurizio.rosati18@outlook.com (M.R.)

**Keywords:** ureteral endometriosis, laparoscopic ureteral reimplantation, robotic-assisted ureteral reimplantation, deep infiltrating endometriosis, ureteral obstruction treatment

## Abstract

**Introduction:** Endometriosis, characterized by the presence of endometrial tissue outside the uterus, includes deep endometriosis (DE), which can affect the urinary tract. Ureteral endometriosis (UE) is a rare but significant manifestation that can lead to ureteral obstruction, hydronephrosis, and potential kidney loss. This systematic review evaluates the effectiveness and outcomes of laparoscopic versus robotic-assisted ureteral reimplantation techniques in patients with UE. **Materials and Methods:** A systematic literature search was conducted following PRISMA guidelines across PubMed, MEDLINE, Embase, Web of Science, and the Cochrane Library, from inception to July 2024. Studies included patients with UE who underwent ureteral reimplantation using laparoscopic or robotic-assisted techniques. Data on patient demographics, surgical technique, duration of surgery, complications, follow-up duration, and clinical outcomes were extracted and analyzed. **Results:** Twelve studies met the inclusion criteria, comprising 225 patients in the laparoscopic group and 24 in the robotic-assisted group. Lich–Gregoir ureteral reimplantation, with or without a psoas hitch, was the predominant technique used. The average surgery duration was 271.1 min for the laparoscopic group and 310.4 min for the robotic-assisted group. Recurrence rates for UE were 2.95% for laparoscopic and 5.9% for robotic-assisted procedures. The robotic-assisted group had a significantly shorter hospital stay (6.7 days vs. 9.1 days, *p* < 0.01). Postoperative complication rates were comparable between the two techniques (*p* = 0.422). **Conclusions:** Both laparoscopic and robotic-assisted techniques for ureteral reimplantation in UE are safe and effective, with the choice of technique guided by surgeon expertise and specific clinical scenarios. However, the limited number of robotic cases introduces a bias, despite statistical significance.

## 1. Introduction

Endometriosis, characterized by endometrial glands and stroma outside the uterus, is classified into three forms: superficial or peritoneal, ovarian, and deep endometriosis (DE) [1]. Endometriosis, in its various phenotypes, is now a condition that can be surgically addressed using a range of safe and effective minimally invasive techniques [2,3]. DE of the urinary tract varies with an incidence of 0.3% to 12%, resulting in frequent extragenital site disease localization [4]. Ureteral endometriosis (UE) is a rare but significant manifestation of endometriosis, affecting approximately 0.1% to 1% of patients, with a left predisposition in most cases, confined to the distal segment of the ureter at 3 to 4 cm above the vesicoureteral junction [5,6]. The clinical presentation of UE can be challenging to diagnose due to the absence of specific symptoms in many cases. UE is often associated with other sites of DE [7]. Consequently, the symptoms are frequently attributable to these other locations rather than ureteral endometriosis alone. Indeed, when managing endometriosis overall, especially for cases presenting solely with infertility, there is no absolute consensus on when to opt for surgical treatment versus assisted reproductive technologies [8]. However, when managing ureteral endometriosis and there are indirect signs of renal insufficiency due to obstruction, the priority is to restore renal function over other considerations. Therefore, UE is a threatening localization of disease burden and is characterized by the presence of endometrial tissue within or surrounding the ureter, leading to potential complications that can evolve into urinary tract obstruction with subsequent hydronephrosis and potential kidney loss [9]. Histopathologically, two major types of endometriosis are recognized according to the grade of infiltration of the ureteral wall: intrinsic and extrinsic [10]. In the intrinsic type, the ectopic endometrial tissue is found in the muscularis of the ureteral wall, while UE is considered extrinsic when DE lesions are responsible for a significant ureteral obstruction but without involvement of the ureteral muscularis. The two types may occur simultaneously [10,11]. In about one-third of cases, urinary stasis caused by hydronephrosis promotes the development of infections, especially in the upper urinary tract [12]. Radiological imaging techniques such as transvaginal ultrasound, magnetic resonance imaging (MRI), and multislice computed tomography are crucial for the accurate diagnosis of UE [13]. While there have been reports of successful medical treatment outcomes, these interventions are generally insufficient to address the fibrotic nature of the lesions [14,15]; consequently, surgical intervention is typically the primary approach, especially to free ureteral obstruction and prevent further deterioration of renal function, as relying solely on medical therapy carries the risk of treatment failure and worsening of the condition [16]. Surgical options include conservative ureterolysis or more radical procedures such as ureterectomy with end-to-end anastomosis or ureteroneocystostomy, chosen based on the type, location, and extent of ureteral involvement [17]. Ureterolysis, which involves the dissection and release of the ureter from surrounding endometrial tissue, is a common initial approach that should be reserved for patients with no or mild ureter obstruction [18]; this technique provides lower peri- and postsurgical complication rates but higher recurrence of the disease [19]. Ureterolysis alone can result in ischemia of the ureter and urinary fistula postoperatively with the formation of urinomas [20]. The first case of laparoscopic endometriosis ureteral reimplantation was described by C. Nezhat et al. in 1992 [21]. Subsequently, this surgical approach has been consolidated and widely demonstrated to be superior when performed through minimally invasive surgery over laparotomy, particularly in terms of reduced postoperative analgesic requirements, shorter hospitalization, and faster convalescence [22,23]. Robotic-assisted laparoscopic techniques have proven useful in the treatment of extensive urinary tract endometriosis [24]. Conventional laparoscopy and robotic-assisted laparoscopy are excellent methods for treatment of advanced stages of endometriosis. However, the use of the robotic platform may increase operative time and financial costs and might also be associated with a longer hospital stay [25]. The optimal surgical approach to manage ureteral endometriosis (UE) is not clearly defined due to the lack of prospective randomized trials, which are challenging to conduct because of the rarity of the disease. Most available studies are retrospective, uncontrolled, and involve heterogeneous populations.

This systematic review seeks to assess the efficacy and results of various surgical methods for ureteral reimplantation in individuals with endometriosis, emphasizing a comparison between laparoscopic and robotic-assisted techniques to identify the most effective surgical approach.

## 2. Materials and Methods

### 2.1. Literature Search

This review was performed according to the Preferred Reporting Items for Systematic Review and Meta-analysis (PRISMA) statement [26]. No institutional review board approval was required because only published, de-identified data were analyzed. A systematic electronic database search of all published studies, limited to the English language, on endometriosis ureteral reimplant from inception to July 2024 was performed in PubMed, MEDLINE, Embase, Web of Science, and the Cochrane Library. We used combinations of the following Mesh keywords in the search: “Endometriosis”, “Deep Endometriosis”, “Ureteral Endometriosis”, “Ureteral Obstruction”, “Ureteral Reimplantation”, “Ureterolysis”, “Ureteroneocystostomy”, “Hydronephrosis”, and “Double-J Stents” were combined with “Surgery”, “Minimally Invasive Surgical Procedures”, “Robotic Surgical Procedures”, and “Laparoscopy”.

### 2.2. Studies Eligibility

In our study, we included and extracted data only from patients diagnosed with intrinsic or extrinsic ureteral endometriosis who underwent ureteral reimplantation performed by laparoscopy or robotic-assisted laparoscopy (via ureteroneocystostomy or end-to-end ureteroureteral anastomosis). We examined the intraoperative complications, focusing on their frequency and types that occurred during the surgical procedures. Moreover, postoperative complications were also categorized according to the Clavien–Dindo classification [27] to ensure a standardized assessment of their severity and impact, and a sub-analysis was performed to evaluate Clavien–Dindo Grade III and higher complications, given their severity and clinical relevance. In some studies, postoperative complications were reported globally for the entire surgical procedure, without distinguishing between complications directly related to the ureteral reimplantation technique and those from other procedures performed during the same surgery. Where possible, we focused on complications directly associated with the ureteral reimplantation, excluding those related to different procedures. However, in certain cases, it was necessary to consider the reported complications as a whole, recognizing the possibility that some might be related to other aspects of the surgery. Secondary outcomes encompassed a range of additional factors that provided further insights into the efficacy and safety of the surgical techniques. These included the reintervention rate, which indicated the need for additional surgical interventions related to the initial ureteral procedure, and the recurrence rate, representing the percentage of patients experiencing a recurrence of ureteral endometriosis or related symptoms after surgery. We also considered the duration of hospital stay as an important measure of recovery time, along with the follow-up duration, which reflected the duration for which patients were observed following surgery to evaluate long-term outcomes and complications. These primary and secondary outcomes collectively allowed us to determine the overall efficacy and safety of the different surgical approaches for ureteral reimplantation, comparing laparoscopic and robotic surgical methods, and identifying best practices for managing ureteral endometriosis. Preoperative indications across different studies revealed that ureteral endometriosis with associated hydronephrosis was the most common indication for surgery. The decision for intervention was primarily driven by imaging findings showing ureteral stenosis and renal impairment, as well as symptoms of pain in some cases. Notably, some patients presented with silent renal dysfunction, highlighting the need for thorough preoperative assessment. In our systematic review, we focused on extracting specific parameters from the selected studies, including the first author and year of publication, study design, total number of patients, mean age, type of endometriosis (intrinsic or extrinsic to the ureter as reported in the studies), surgical technique used (e.g., ureteroneocystostomy, end-to-end ureteroureteral anastomosis), mean duration of the surgical procedures, intraoperative complications, reintervention for ureteral reimplantation complications, postoperative complications (using the Clavien–Dindo classification), mean follow-up duration, days of hospital stay, recurrence rates of endometriosis ureteral obstruction, and overall clinical outcomes. We included studies that provided detailed information on ureteral reimplantation techniques in the context of ureteral endometriosis and reported on most of the key parameters listed above. We excluded studies that did not specify the number of patients who underwent ureteral reimplantation or lacked detailed outcome data specific to ureteral endometriosis, as well as studies that only provided minimal information such as the number of patients and general surgical outcomes without further detail.

### 2.3. Study Selection and Data Extraction

After an initial screening of titles and abstracts retrieved by the search, the full texts of all potentially eligible studies were fully assessed. Only peer-reviewed papers in English were included. The references of the included articles were also reviewed, and additional studies were added if relevant. If there were several publications on the same patient series developing over the years, only the latest was included. Studies were also excluded if data from the same or similar series were reported repeatedly. The full texts were examined for eligibility, and articles satisfying the abovementioned criteria were selected.

### 2.4. Objectives

This systematic review evaluates published data on the effectiveness and outcomes of laparoscopic versus robotic-assisted ureteral reimplantation techniques for patients with UE. The outcomes of this review include surgical success in resolving ureteral endometriosis and restoring ureteral function, complication rates categorized by severity, the frequency of reinterventions, recurrence rates of endometriosis-related ureteral obstruction, and the duration of hospital stay. Restoration of ureteral function was assessed based on postoperative imaging studies, such as ultrasound or computed tomography (CT), showing resolution of hydronephrosis or hydroureter. Additionally, renal function improvement was considered using serum creatinine or other renal markers when available.

### 2.5. Statistical Analysis

Descriptive statistics summarized the extracted data, presenting means with standard deviation. To compare complication rates and hospital stay durations between the two surgical approaches, chi-square tests or Fisher’s exact tests were used for categorical variables, and t-tests or Mann–Whitney U tests were used for continuous variables. A *p*-value < 0.05 was considered statistically significant.

## 3. Results

**Figure 1:**
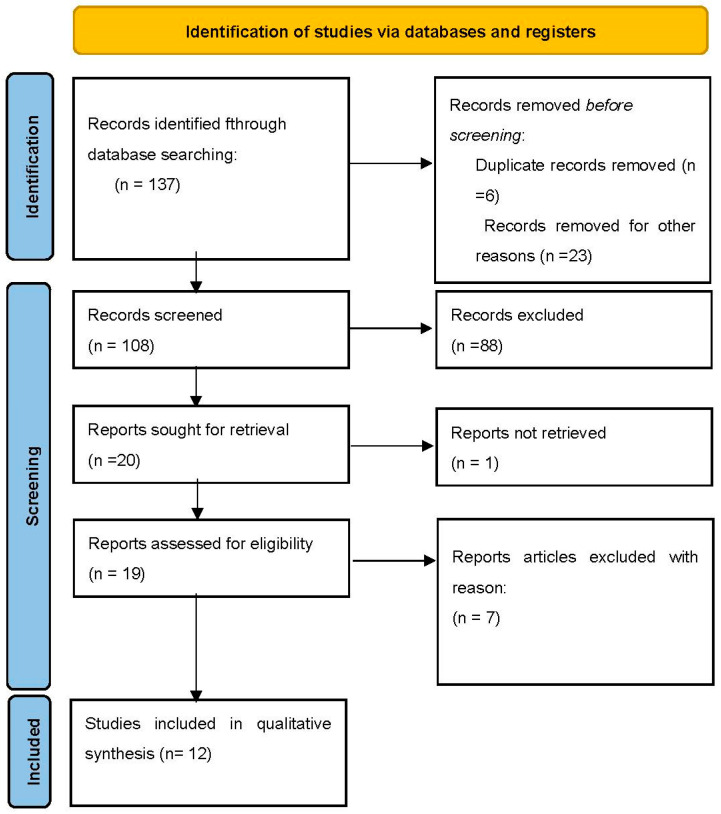
Flow diagram of study identification and selection.

The literature search (Figure 1), based on our predefined key search items, identified 137 publications. Finally, a total of 12 studies were included in the present systematic review. All included publications are retrospective studies except for one prospective study. The selection and elimination of the articles are detailed in the flow chart in Figure 1.

A total of nine studies, including one prospective and eight retrospective studies, were reviewed, encompassing 225 patients in the laparoscopic group with a mean age of 34.8 years and 24 patients in the robotic-assisted group with a mean age of 33.9 years. The majority of procedures were Lich–Gregoir ureteral reimplantations, with or without a psoas hitch, and a mean surgery duration of 271.1 min in the laparoscopic group and 310.4 min in the robotic-assisted group. In none of the included articles were significant intraoperative complications reported, except for two cases in the laparoscopic group where conversion to laparotomy was necessary due to technical difficulties during the procedure. Reintervention for ureteral procedure complications occurred in 3.11% of cases in the laparoscopic group and 4.1% in the robotic-assisted group. Postoperative complications in the laparoscopic group were distributed as follows: Grade I (4.4%), Grade II (12%), and Grade IIIa/b (5.3%). In the robotic-assisted group, postoperative complications included Grade II (11.1%) and Grade IIIa/b (11.1%). A chi-square test showed no significant difference in the overall complication rates between the two techniques (*p* = 0.422). Our sub-analysis focused on Clavien–Dindo Grade III or higher complications and found 14 cases in the laparoscopic group (6.2%) and 1 case in the robotic-assisted group (4.2%). Statistical comparison using a chi-square test did not show a significant difference between the two groups (*p* = 1). The average follow-up period was 22.56 months in the laparoscopic group and 30 months in the robotic-assisted group, with recurrence rates of 2.95% and 5.9%, respectively, with no statistically significant difference (*p* = 1). The average hospital stay was significantly shorter for the robotic-assisted procedures compared to the laparoscopic procedures (6.7 days vs. 9.1 days, respectively, *p* < 0.01). Detailed results are shown in Table 1 and Table 2. Table 3 shows the outcomes and success of each study included in our analysis.

## 4. Discussion

This systematic review aimed to evaluate the safety and effectiveness of laparoscopic and robotic-assisted ureteral reimplantation techniques in the treatment of ureteral endometriosis. Our analysis focused on resolving ureteral obstruction, postoperative complications, and recurrence rates across the included studies. The findings suggest that both laparoscopic and robotic-assisted approaches are effective in restoring ureteral function and resolving symptoms in patients with ureteral endometriosis. The overall length of the procedures was often influenced by the involvement of endometriosis in other anatomical sites, which required treatment alongside the ureteral reimplantation. Therefore, it is not appropriate to attribute the total surgical time exclusively to the ureteral reimplantation technique. As a result, inferential statistics for operative time were not performed. Similarly, blood loss data were not analyzed due to the presence of concurrent procedures (e.g., intestinal resections) during many of the surgeries, making this parameter unreliable for comparison. The study by Ceccaroni et al. [28], the largest retrospective cohort in this review, provided the only detailed distinction between the total surgery duration and the specific time dedicated to ureteral reimplantation. Furthermore, the study identified cases where endometriosis recurred in the parametrial region, leading to a new ureteral obstruction after the initial surgery. Reintervention was required in these patients, and histological examination confirmed the presence of endometriosis, establishing this as a recurrence of the disease. In some instances, the recurrence occurred on the contralateral side, rather than in the area previously operated, further highlighting the unpredictable nature of the disease’s progression.

### 4.1. Outcome Analysis of Laparoscopic and Robotic-Assisted Surgery

#### 4.1.1. Hospital Stay and Duration of Surgery

Robotic-assisted procedures were associated with a significantly shorter hospital stay, with an average of 6.7 days compared to 9.1 days for laparoscopic procedures (*p* < 0.001). This shorter hospitalization period can be attributed to the minimally invasive nature of robotic surgery, which generally results in less postoperative pain and faster recovery times. In a comparison study of analgesic requirements in robot-assisted versus conventional laparoscopic abdominal surgery procedures, the analgesic requirements were significantly less in robot-assisted laparoscopic surgery in the first 24 h [40]. Some studies, such as Kawka et al. [41], have highlighted potential benefits of robotic-assisted surgery, including shorter recovery times and better patient-reported outcomes, but these findings remain inconsistent. Robotic-assisted surgeries generally require more time, largely due to the additional docking time associated with the robotic system and higher costs. As already reported in a national analysis of cost disparities in robotic-assisted versus laparoscopic abdominal operations [42], despite the benefits of reduced hospital stay, the overall cost-effectiveness of robotic-assisted surgery is warranted to justify its greater costs and remains a topic for further investigation.

#### 4.1.2. Recurrence of Endometriosis-Related Ureteral Obstruction

Our review shows that while laparoscopic procedures had lower recurrence rates compared to robotic-assisted techniques (2.95% vs. 5.9%, respectively), this difference was not statistically significant.

#### 4.1.3. Complication Comparison between Laparoscopic and Robotic-Assisted Surgery

Two significant surgical complications were reported in the reviewed articles, both occurring in the laparoscopic group and requiring conversion to laparotomy. Postoperative complications were largely minor, with most categorized as Clavien–Dindo Grades I and II. However, there were isolated cases of Grade III complications, which were more prevalent in the robotic-assisted group, though the difference was not statistically significant. This sub-analysis of Grade III and higher complications showed no significant difference between the two surgical approaches, further supporting the fact that both are safe options for ureteral reimplantation. The chi-square test for independence revealed no significant association between the type of surgery and the occurrence of postoperative complications (*p* = 0.422). This indicates that both laparoscopic and robotic-assisted techniques are comparable in terms of complication rates. However, given the variability in reporting across the studies and the inclusion of multiple concurrent procedures in some cases, these findings must be interpreted with caution.

### 4.2. Surgical Techniques for Ureteral Reimplantation

The Lich–Gregoir technique was the most employed method for ureteral reimplantation across the studies, favored for its simplicity, safety, effectiveness in preventing vesicoureteral reflux, and high success rate, which can exceed 95% [43,44]. The Boari flap was utilized when more extensive ureteral defects made the Lich–Gregoir method impractical. The Boari flap, sometimes combined with a psoas hitch, has also been shown to be a safe and effective method for treating complex ureteral defects [45]. The Boari flap is often combined with a psoas hitch to ensure tension-free anastomosis and is a preferred method for complex distal ureteral defects when the ureteral segment is too short to reach the bladder without tension [46].

## 5. Conclusions and Limitations

This systematic review supports the safety and efficacy of both laparoscopic and robotic-assisted ureteral reimplantation for treating ureteral endometriosis. The findings indicate that while the recurrence rate appears to be equal between the two groups, robotic-assisted techniques offer a significantly shorter hospital stay. The observed complication rates are low and comparable between the two approaches, reinforcing the viability of both minimally invasive techniques. The choice between laparoscopic and robotic-assisted methods should be tailored to the surgeon’s expertise and the specific clinical circumstances of each patient. The limitations of this systematic review include the retrospective nature of most included studies and the heterogeneity in study populations and methodologies, making it difficult to establish whether both cohorts were fully comparable in terms of surgical risk and complexity; indeed, such data were not consistently available across the studies included in the systematic review. Additionally, the significant difference in sample sizes between the two groups introduces potential bias, with 225 patients in the laparoscopic cohort compared to only 24 in the robotic-assisted group. This disparity means that the statistical significance of some results should be interpreted with caution. Furthermore, the lack of standardized reporting on specific surgical outcomes, such as intraoperative and postoperative complications categorized by the Clavien–Dindo classification, limits the comparability of results across studies. The presence of concurrent endometriosis at other sites often influenced the duration of surgery and postoperative recovery, complicating the attribution of outcomes solely to the ureteral re-implantation procedure. Another limitation of this review is that most studies did not consistently report specific thresholds for renal function improvement, which limits the ability to standardize the measurement of ureteral function restoration across studies. Further prospective, randomized controlled trials with larger sample sizes are needed to confirm these findings and better define the optimal surgical approach for ureteral endometriosis.

## Figures and Tables

**Table 1:** The main characteristics of laparoscopic surgery considered studies with complications, recurrence, and hospital stay.

Author and Year	Study Type	N. of Patients	Mean Age (Years, Mean)	Type of Endometriosis (Intrinsic/Extrinsic)	Surgical Technique	Duration of Surgery (Min-Mean)/and Ureteral Reimplantation (Mean)	Intraoperative Complications	Reintervention for Ureteral Reimplantation Complications	Postoperative Complications (Clavien–Dindo Grading System for Surgical Complications)	Follow-Up (Months)	Recurrence Rate (%)	Time of Hospitalization (Days, Mean) SD
Ceccaroni et al., 2018 [28]	Prospective study	160	36.1	Intrinsic/ Extrinsic	Laparoscopic Ureteroneocystostomy 160/160 (100%) Lich–Gregoir or direct reimplantation with or without psoas hitch	364.3/92.3 (120–600/30–180)	None	3 bladder suture leakage (1 with associated pelvic abscess, 1 hemoperitoneum)	Grade I: 6 (3.7%) Grade II: 12 (7.5%) GradeIIIb: 7 (4.4%)	>6 months	1.2%	8 (7–18)
Schonman et al., 2013 [29]	Retrospective	1	34.3	Not specified	Not specified	Not specified	2 laparotomy conversion after laparoscopic attempt	None required	None	63	0%	Not specified
Bourdel et al., 2015 [30]	Retrospective	3	32	Extrinsic	Lich–Gregoir	226.67 (120–480)	None	None required	Grade III: 1 (33.3%)	22.5	0%	12.6 (6–26)
Chudzinski et al., 2017 [31]	Retrospective	3	28	Not Specified	Psoas-hitch 70%, 3 vescical bipartition and 1 boari flap	300 (174–426)	None	1	Grade IIIa: 1 (33.3%)	48	Not specified	10.2 (4–16)
Alves et al., 2017 [32]	Retrospective	13	32.1	Intrinsic/ Extrinsic	18 end-to-end anastomosis and 1 ureteral reimplantation (boari flap)	157 (90–330)	Not specified	1 (reimplantation after failed reanastomosis)	Grade II: 1 (7.6%) Grade IIIb: 2 (15.3%)	2 months (longer follow-up was incomplete)	4 (19%) but includes also 8 patients who underwent ureterolysis	Not specified
Stepniewska et al., 2010 [33]	Retrospective	20	35	Intrinsic/ Extrinsic	Lich–Gregoir or Boari flap when necessary	313 (120–500)	Not specified	None required	Grade I: 4 (20%) Grade II: 10 (50%) Grade IIIa: 1 (5%)	6 months	Not specified	10 (7–17)
Ahn et al., 2013 [34]	Retrospective	2	49.5	Not specified	Lich–Gregoir with or without psoas hitch	137 (104–228)	Not reported	None required	None	12	0%	7 (7–7)
Azioni et al., 2010 [35]	Retrospective	6	33.6	Intrinsic/ Extrinsic	Lich–Gregoir	320 (250–440)	none	None required	None	none		8.3 (7–10)
Mereu et al., 2010 [36]	Retrospective	17	32.7	Not specified	17 end-to-end ureteral anastomosis	330 (60–540)	None	2 persistent ureteral stenosis requiring further intervention of ureteroneocystostomy	Grade II: 4 (23.5%)	21	12.5%	8 (2–31)
Total	8 retrospective 1 prospective studies	225 patients	34.8	/	Lich–Gregoir ureteral reimplantation was the preferred technique with or without psoas-hitch	271.1	2 laparotomy conversion	7/225 (3.11%)	Grade I: 10 (4.4%) Grade II: 27 (12%) Grade IIIa/b: 12 (5.3%)	22.56	2.95%	9.1

**Table 2:** The main characteristics of robotic-assisted laparoscopic surgery considered studies with complications, recurrence, and hospital stay.

Author and Year	Study Type	N. of Patients	Mean Age (Years, Mean)	Type of Endometriosis (Intrinsic/Extrinsic)	Surgical Technique	Duration of Surgery (Min)/and Ureteral Reimplantation (Mean)	Intraoperative Complications	Reintervention for Ureteral Reimplantation Complications	Postoperative Complications	Follow-Up (Months, Mean)	Recurrence Rate (%)	Hospital Stay (Days, Mean)
Chudzinski et al., 2017 [31]	Retrospective	4	31	Not Specified	Psoas-hitch 70%, 3 vescical bipartition and 1 boari flap	321 (174–426)	None	1 (25%)	Grade IIIb: 1 (25%) Grade II: 1 (25%)	48	15%	10.2 (4–16)
Yang et al., 2011 [37]	Retrospective	1	28	Not specified	Distal ureterectomy with psoas hitch	Not specified	None	None	none	24	none	4 (4–6)
Hung et al., 2020 [38]	Retrospective	4	36.2	Not Specified	Terminoterminal ureteral anastomosis, ureteroneocystostomy	299.8 (220–404)	None	None	None	17	none	8.6 (7–11)
Di Maida et al., 2020 [39]	Retrospective	15	34.7	Not specified	13 Lich–Gregoir ureteral reimplantation with psoas hitch, 2 end-to-end anastomosis	Not specified	Not specified for the subgroup	None	Not specified for the subgroup	31.3	8.7%	4 (4–6)
Total	4 retrospective studies	24 patients	33.9	/		310.4	None	1/24 (4.1%)	Grade III: 1/9 (11.1%) Grade II: 1/9 (11.1%)	30	5.9%	6.7

**Table 3:** Clinical Outcomes and Restoration of Ureteral Function Following Ureteral Reimplantation in Ureteral Endometriosis.

Study	Resolution of Symptoms	Restoration of Ureteral Function	No. of Patients with Ureteral Function Improvement
Ceccaroni et al., 2018 [28]	Improvement or complete resolution of pain and urinary symptoms in most patients.	Achieved effective drainage of the kidney without obstruction, confirmed through follow-up imaging studies.	160/160 (100%); resolution of hydronephrosis observed on postoperative CT scans and ultrasounds.
Schonman et al., 2013 [29]	Significant improvement in pain and urinary symptoms.	Postoperative imaging confirmed effective ureteral function without obstruction.	1/1 (100%); postoperative ultrasound confirmed no ureteral obstruction or hydronephrosis.
Bourdel et al., 2015 [30]	Improvement in pain and urinary symptoms for most patients.	Effective drainage of the kidney, confirmed via imaging studies.	3/3 (100%); improvement in hydronephrosis based on postoperative ultrasound.
Chudzinski et al., 2017 [31]	A notable improvement in symptoms was observed.	Postoperative imaging indicated successful ureteral function	3/4 (75%); resolution of hydronephrosis on postoperative CT scans and ultrasounds, creatinine levels were monitored in the early postoperative period.
Alves et al., 2017 [32]	Patients reported reduced pain and better urinary function.	Imaging studies confirmed effective kidney drainage without obstruction.	13/13 (100%); resolution of hydronephrosis confirmed by CT and ultrasound.
Stepniewska et al., 2010 [33]	Significant reduction in symptoms for most patients.	Effective ureteral function confirmed by imaging	19/20 (95%); postoperative CT and ultrasound indicated resolution of hydronephrosis.
Ahn et al., 2013 [34]	Improvement in pain and urinary symptoms.	Postoperative imaging showed effective kidney drainage.	2/2 (100%) of patients, hydronephrosis resolved based on follow-up ultrasound and CT scans.
Azioni et al., 2010 [35]	Most patients experienced symptom relief.	Effective ureteral function as indicated by follow-up imaging.	6/6 (100%); resolution of ureteral obstruction observed on follow-up ultrasound.
Mereu et al., 2010 [36]	Improvement in symptoms was observed.	Postoperative imaging confirmed effective ureteral function.	15/17 (88%); follow-up ultrasound and CT scans showed improvement in hydronephrosis
Yang et al., 2011 [37]	Significant symptom reduction.	Imaging studies confirmed effective ureteral function.	1/1 (100%); postoperative ultrasound and CT scans showed resolution of hydronephrosis.
Hung et al., 2020 [38]	Patients reported improvement in pain and urinary symptoms	Effective kidney drainage was confirmed through imaging	4/4 (100%); hydronephrosis resolved as per postoperative CT and ultrasound findings.
Di Maida et al., 2020 [39]	Notable symptom relief was observed.	Postoperative imaging indicated successful ureteral function.	13/15 (87%), follow-up CT and ultrasound showed restored ureteral function.

## Data Availability

Data are available under request to the corresponding author.
